# Placental inflammation is increased in gestational diabetes mellitus: The role of inflammasome NLRP-3 and chemokine scavenger decoy receptor D6

**DOI:** 10.1371/journal.pone.0326087

**Published:** 2025-06-17

**Authors:** Marianna Onori, Giuliana Beneduce, Filomena Colella, Donatella Lucchetti, Caterina Policola, Vincenzo Arena, Fabio Sannino, Alessandro Petrecca, Dario Pitocco, Alfredo Pontecorvi, Alessandro Sgambato, Giovanni Scambia, Nicoletta Di Simone, Tullio Ghi, Chiara Tersigni

**Affiliations:** 1 Università Cattolica del Sacro Cuore, Rome, Italy; 2 Fondazione Policlinico Universitario A.Gemelli IRCCS, Rome, Italy; 3 Humanitas University, Milan, Italy; 4 IRCCS Humanitas Research Hospital, Milan, Italy; National Institute of Pharmaceutical Education and Research Guwahati, INDIA

## Abstract

**Background:**

Gestational diabetes mellitus is characterized by low-grade systemic inflammation. Placental inflammation in gestation diabetes mellitus has not been extensively investigated yet.

**Objectives:**

Aims of this study were to analyze: a) serum levels of Th-1 cytokines and D6-specific chemokines in women with gestation diabetes mellitus, compared to normal pregnant women; b) placental expression of the inflammasome NLR family pyrin domain containing 3 (NLRP-3) and the chemokines scavenger decoy D6 receptor.

**Methods:**

Serum samples collected between 24 and 28 weeks of pregnancy from singleton pregnancies with gestational diabetes mellitus and gestational age-matched normal pregnant women were analyzed by bead-based multiplex assays for chemokine (C-C motif) ligand 2 (CCL2), chemokine (C-C motif) ligand 4 (CCL4), interferon gamma (IFN-γ), C-C motif chemokine ligand 11 (CCL11) and tumor necrosis factor alpha (TNF-α) levels. Placental samples from GDM and controls were analysed by immunohistochemistry and multiplex spatial immunofluorescence for protein expression of NLR family pyrin domain containing 3 (NLRP-3), interleukin-1 beta (IL-1β) and chemokines scavenger decoy D6 receptor.

**Results:**

GDM women (n = 25) showed higher serum levels of CCL-2 (p < 0.01), CCL-4 (p < 0.05) and IFN-γ (p < 0.05) compared to controls (n = 25). Placental expression of NLRP-3 was significantly higher in GDM women (n = 10) compared to controls (n = 7; p < 0.05) while only a trend of increase of IL-1β and D6 expression was observed in GDM compared to normal placentas.

**Conclusions:**

GDM is characterized by higher serum levels of pro-inflammatory cytokines with consistent over-expression of the inflammasome NLRP-3 in placental tissues compared to normal pregnancy.

## 1. Introduction

Gestational diabetes mellitus (GDM) is among the leading complications of pregnancy with a global prevalence of 14% of all pregnancies [1]. GDM can lead to severe obstetric complications such as preterm birth, intrauterine death, fetal macrosomia and dystocia, [2–4], as well as increased risk for mother and child of developing type II diabetes and metabolic syndrome during life [5]. In the last decades, the incidence of GDM has steeply increased because of the concomitant increase of maternal age and BMI at conception. Obesity plays a major role in the pathophysiology of GDM which is generally characterized by excessive maternal peripheral insulin resistance, increased systemic inflammation, higher circulating levels of free fatty acids (FFAs) and advanced glycation end products (AGEs). Interestingly, women with GDM show increased serum [6] and adipose tissue [7] inflammation. In particular, higher serum levels of IL-6 and TNF-alpha and increased concentrations of IL-1β in adipose tissues have been showed in GDM women [6,7]. However, the role of placental inflammation in the development of GDM and related obstetric complications is still matter of investigation. In a preclinical murine model of GDM dampening placental inflammation ameliorates insulin-resistance, endothelial dysfunction and live birth rate [8].

Evidence of the role of placental inflammation in the physiopathology of obstetric disorders has been rapidly growing in the last years [9–11]. Noteworthy, the inflammasome NLR family pyrin domain containing 3 (NLRP-3), abundantly expressed in the human placenta [12], has been studied in preeclampsia [12,13], recurrent pregnancy loss [13] and preterm delivery [11]. The inflammasome NLRP-3 is an intracellular multiprotein complex belonging to the innate immunity, which is activated by several stimuli (i.e., pathogen-associated molecular patterns, i.e., lipopolysaccharides (LPS), danger-associated molecular patterns, reactive oxygen species) [14,15], and finally leads to the release of pro-inflammatory cytokines (IL-1β and IL-18).

The chemokine scavenger decoy receptor D6 also plays a pivotal role in the dysregulation of human placental inflammation and its function has been studied, particularly, in pre-eclampsia [16,17]. Thus, both NLRP-3 and D6 receptor represent key molecules in the control of placental inflammation necessary for a good pregnancy outcome [18,19].

Aims of this study were to evaluate: a) serum levels of pro-inflammatory cytokines and D6-specific chemokines in women with GDM, compared to women with uncomplicated pregnancies matched for gestational age; b) the placental expression of the inflammasome NLRP-3, IL-1β and of decoy receptor D6 in GDM and healthy pregnant women.

## 2. Materials and methods

### 2.1 Study population

This study was conducted according to the principles of the Declaration of Helsinki and approved by the Ethics Committee of the Fondazione Policlinico A. Gemelli IRCCS of Rome (ID:4978 date of approval 9/06/2022). Written informed consent was collected from all patients recruited in the study.

Women with GDM and healthy pregnant women, matched for gestational age, were recruited at diagnosis between 24 and 28 weeks of gestation in a consecutive fashion in the period between 1^st^July 2022 and 30^th^ June 2023 at the High Pregnancy Risk outpatients of the Fondazione Policlinico A. Gemelli IRCCS of Rome, Italy. Diagnosis of GDM was made in case of fasting glucose levels>92 mg/dl or at least one abnormal glucose values at the 75g oral glucose tolerance test (OGTT). Briefly, blood sampling was performed before the glucose load, 60 and 120-minutes post-load. GDM was diagnosed in case of fasting serum glucose levels: ≥ 92 mg/dl, or glucose levels ≥180 mg/dl 60 minutes after load and/or glucose levels ≥153 mg/dl 120 minutes after load [20]. Controls were recruited in a consecutive fashion among normal pregnant women with negative OGTT matched with cases for gestational age. Exclusion criteria were type 1 and 2 diabetes, multiple pregnancies, maternal infectious diseases and autoimmune diseases.

### 2.2 Samples collection

3 ml of venous blood were collected from GDM patients at diagnosis and in gestational-age matched controls via venipuncture from the antecubital fossa. Blood samples were centrifuged at 1200 g for 10 min at 20°C to remove the cellular component and collect the serum that was, then, frozen in 500 μl aliquots at −80°C until further use. Placental biopsies of 2x2 cm were collected from GDM patients and normal pregnant women delivering at term by elective caesarean section, in absence of labor, for obstetrical indications (breech presentation, previous caesarean section). Placental samples were fixed in 10% formalin and embedded in paraffin for the routine histopathological examination and for subsequent immunohistochemical and immunofluorescence analyses. All women recruited in this study delivered in our center and clinical data related to obstetric and neonatal outcomes and pathological examination of the placenta were collected.

### 2.3 Luminex assay

The analysis of serum levels of pro-inflammatory cytokines and D6-specific chemokines was performed using a Human Magnetic Luminex Assay (Bio-Techne R&D system, Minneapolis, Minnesota, USA) for CCL2, CCL4, IFN-γ, IL-6, CCL3, CCL11, IL-1β and TNF-α, according to manufacturer instructions. The concentrations were calculated by software provided by the manufacturer using the standard curve. Serum samples were assayed in duplicate and averaged to calculate concentrations.

### 2.4 Hematoxylin–eosin staining and immunohistochemistry assay

Hematoxylin and eosin (H&E) staining and immunohistochemistry (IHC) were performed on 3 μm sections of FFPE human placental tissue. Sections were dewaxed by successive washes in xylene (5 min each), followed by rehydration in a graded series of alcohol solutions: 100% (twice), 95%, 90%, 80%, 70% and 50%. The sections were then stained with Mayer’s hematoxylin (Bio-Optica Milano S.p.a., catalogue number: 05-M06002) and Eosin Y alcoholic solution (Bio-Optica Milano S.p.a., catalogue number: 05-M10003) according to the manufacturer’s instructions. Immunohistochemistry (IHC) was performed using the Leica BOND RX automated immunostainer (Leica Microsystems, Milton Keynes, UK). Primary antibodies against NLRP3 (clone SC06–23, code MA5–32255, 1:200 dilution, Thermo Fisher), CRD6 (clone B-12, code sc-365718, 1:100 dilution, Santa Cruz) and anti- IL-1 β antibody (dilution 1:100, clone H-153 sc-7884, Santa Cruz, CA, USA) were detected using the BOND Polymer Refine Detection system (DS9800). Images were acquired using the Vectra Polaris™ Automated Quantitative Pathology Imaging System (Akoya Biosciences) at 20 × magnification. Analysis of positive cell intensity and density and calculation of H-score were performed using QuPath software version 0.5.1.

### 2.5 Multiplex immunofluorescence staining

A multiplex fluorescence IHC panel was performed on 3 μm sections of FFPE human placental tissue using antibodies against cytokeratin 7 (mouse monoclonal, OV-TL-12, Dako, Glostrup, Denmark), NLRP-3 (rabbit monoclonal, sc06–23, Invitrogen, Waltham, MA, USA) and CRD6 (mouse monoclonal, sc-365718, Santa Cruz Biotechnology, Inc. Dallas, Texas, USA). Staining was performed on the Leica BOND RX automated immunostainer (Leica Microsystems, Milton Keynes, UK) using the Opal colour IHC kit (PerkinElmer, Waltham, USA, Cat. No. NEL820001KT). The fluorophores and DAPI were used according to manufacturers’ instructions. Images were acquired using Phenoimager HT Imaging system (Akoya Biosciences) at ×20 magnification and processed using inForm software v2.6.0 (PerkinElmer, Inc.).

### 2.6 Statistical analysis

Clinical data are shown as mean ± standard deviation (SD) or percentage (%), depending on type of variables. Data were evaluated for normal distribution using the Shapiro–Wilk test and analyzed by Chi-squared test, Mann–Whitney U-test, Student’s t-test, univariate linear regression as appropriate, using Prism software version 10. For all analyses, *p* < 0.05 was considered significant.

## 3. Results

### 3.1 Characteristics of the study population and obstetric outcomes

25 women with GDM and 25 with uncomplicated singleton pregnancies, matched for gestational age, were recruited for the analysis of serum levels of pro-inflammatory cytokines and D6-specific chemokines. Clinical characteristics of the study population are summarised in Table 1. In the population recruited for serum cytokines analysis (n = 50), a significant higher BMI at booking was found in the GDM group recruited compared to controls (p < 0.05). In contrast, a lower weight gain during pregnancy was observed among GDM women compared to normal pregnant women (p < 0.001). Women with GDM showed lower gestational age at delivery compared to controls. Consistently, a significantly lower birth weight was observed in the GDM group compared to the controls. Clinical characteristics of the study population recruited for placental tissues (n = 17) experiments are summarised in S1 File.

**Table 1:** Clinical characteristics and obstetric outcomes of women recruited in this study for serum.

	GDM (n = 25)	CTR (n = 25)	p
Age (years)	36.4 ± 6.1	33.7 ± 4.5	0.08
BMI at booking (Kg/m^2^)	25.4 ± 6.5	22.7 ± 3.2	**<0.05**
<25	13 (52%)	19 (76%)	0.07
25 ≥ 30	7 (28%)	6 (24%)	0.7
>30	5 (20%)	0 (0%)	0.08
Δelta BMI (Kg/m^2^)*	3.2 ± 2.1	4.2 ± 2.2	0.1
Weight gain (Kg)*	9.3 ± 3.6	12.8 ± 2.3	**<0.001**
Race			
* White*	23 (92%)	24 (96%)	0.5
* Hispanic*	0 (0%)	0 (0%)	–
* Black*	0 (0%)	0 (0%)	–
* South-East Asian*	2 (8%)	1 (4%)	0.5
* *Nulliparous	4 (16%)	4 (16%)	1
* *Multiparous	21 (84%)	21 (84%)	1
* *Previous fetal losses	3 (12%)	0 (0%)	0.3
* *Insulin	12 (48%)	0 (0%)	**<0.01**
* *Diet therapy	13 (52%)	0 (0%)	**<0.0001**
* *GA delivery (weeks)	38.1 ± 1.6	39.6 ± 1.2	**<0.001**
* *Induction of labor	11 (44%)	8 (32%)	0.4
Delivery mode			
* CS*	13 (52%)	9 (36%)	0.2
* Vaginal delivery*	12 (48%)	16 (64%)	0.2
* *Neonatal Weight (g)	3051.8 ± 514.7	3387.7 ± 390.9	**<0.05**
* *Percentile (°)	53.4 ± 26.6	57.8 ± 28.1	0.6

Data are expressed as mean ± SD or percentage, according to variables. % refers to the whole cohort of women. BMI: body mass index; GA: gestational age.*BMI or weight gain refers to the time interval from booking to the term of pregnancy. CS: caesarean section.

### 3.2 GDM women showed higher serum levels of IFN-γ, CCL-2 and CCL-4

**Fig 1:**
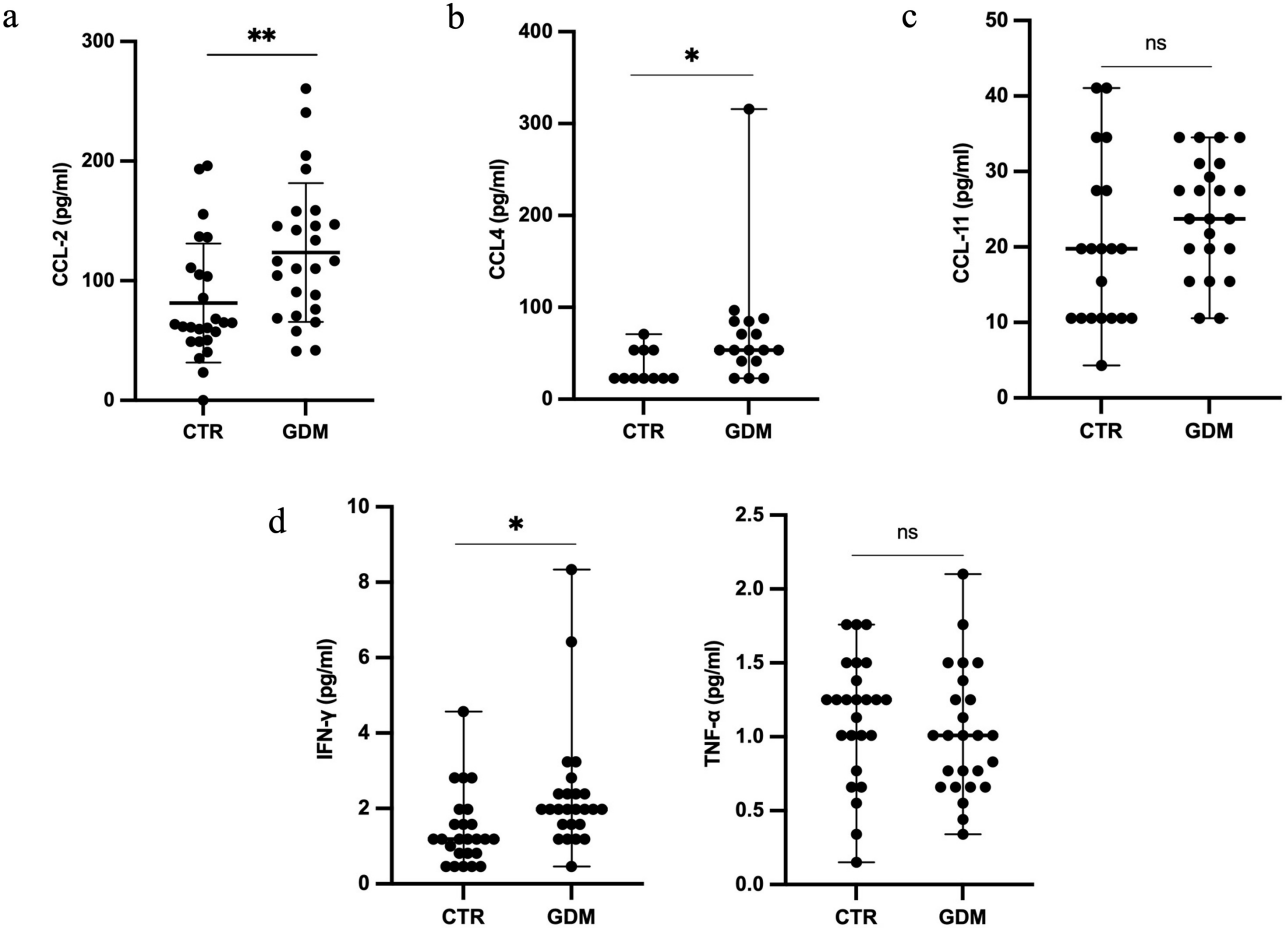
a-e: Scatter plot representing serum levels of CCL-2 (a), CCL-4 (b), CCL-11 (c), IFN-γ (d) and TNF-α (e) in GDM women (n = 25) compared to controls (n = 25). Results are expressed as mean ± SD (a, e) or median (b,c,d), according to variables distribution. GDM: gestational diabetes mellitus; CTR: controls; IFN-γ: interferon-γ; CCL: chemokine ligands; TNF-α:tumor necrosis factor-alfa; ns: not significant; *p < 0.05; **p < 0.01.

Higher serum levels of the IFN-γ and pro-inflammatory chemokine CCL-2 and CCL-4 were observed in GDM women compared to normal pregnant women (Fig 1 a, b, d). No significant differences in serum levels of chemokines CCL-11 and TNF-α were observed between GDM women and controls (Fig 1c and e). Serum levels of interleukin-6 (IL-6), interleukin-1beta (IL-1β) and chemokine CCL-3 were found out of the detectable range (data not shown).

### 3.3 Correlation between systemic inflammation and BMI in GDM

**Fig 2:**
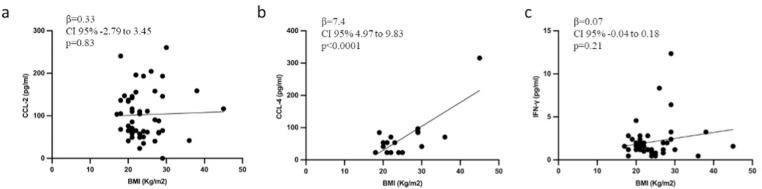
a-c: Scatter plot representing the correlation between serum CCL-2 (a), CCL-4 (b) and IFN-γ (c) levels with maternal BMI. IFN-γ: interferon-γ; CCL: chemokine ligands; BMI: body mass index.

Linear univariate analysis of the whole study population (n = 50) did not show a significant correlation between serum levels of CCL-2 or *IFN-γ* and maternal BMI (β = 0.33, CI 95% −2.79 to 3.45, p = 0.83 and β = 0.07, CI 95% −0.04 to 0.18, p = 0.21, respectively; Fig 2 a and c). Maternal BMI was correlated only to serum levels of CCL-4 (β = 7.4, CI 95% 4.97 to 9.83, p < 0.0001; Fig 2b).

### 3.4 GDM placentas showed higher NLRP-3 expression

**Fig 3:**
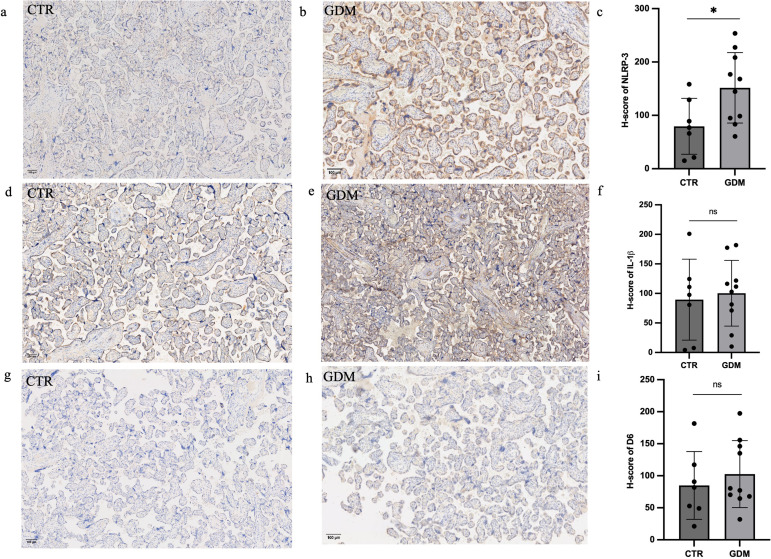
a-b, d-e, g-h: Representative micrographs of immunohistochemical staining for NLRP-3 (a,b), IL-1β (d,e) and *D6 (g,h) of placentas from normal pregnant women (a,d,g) and GDM women (b,e,h). c,f and i: Histograms representing placental expression of NLRP-3 (c)* IL-1β *(f) and D6 (i) in GDM cases and controls. CTR: controls; GDM: gestational diabetes mellitus; NLRP-3:* NLR family pyrin domain containing 3; IL-1β: interleukin 1 beta; CRD6: chemokine scavenger decoy receptor D6. Data are expressed as mean±SD. *p < 0.05; ns = not significant; (Scale Bar: 100 µm).

The expression of the inflammasome NLRP-3 and the chemokine scavenger decoy receptor D6 was assessed in placentas of GDM (n = 10) and normal pregnant (n = 7) women, matched for gestational age (S1 File). Increased placental expression of NLRP-3 was observed by IHC in women with GDM compared to controls (p < 0.05) (Fig 3a-c). Placental expression of IL-1β and D6 decoy receptor was slightly higher in GDM placentas compared to controls, although not reaching the statistical significance (Fig 3d-i; p = 0.7, p = 0.2, respectively).

**Fig 4:**
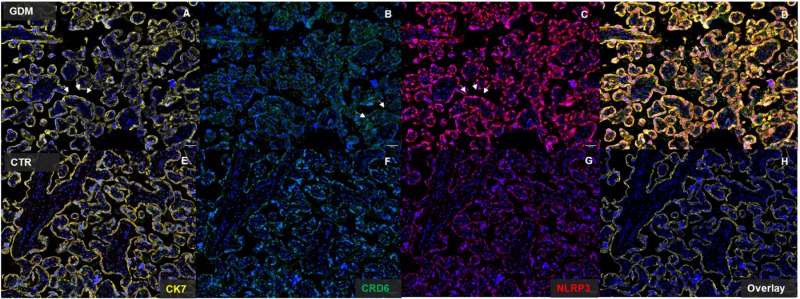
Immunofluorescence staining of CK7, NLRP-3, D6 and DAPI in placenta of GDM woman (A-D) and of normal pregnant women (E-H). (A, E) CK7 (yellow); (B, F) D6 (green); (C,G) NLRP-3 (red); (D, H) Merge. CK7: cytokeratin 7; NLRP-3: NLR family pyrin domain containing 3; CRD6: chemokine scavenger decoy receptor D6; DAPI (blue): 4′,6-diamidino-2-phenylindole.

The immunofluorescent staining confirmed the higher expression of both the NLRP-3 and the D6 decoy receptor, providing information on the tissue localization and distribution of the proteins of interest. In particular, CK7 and NLRP-3 showed a characteristically membrane distribution in the syncytiotrophoblast of the placenta villi. On the other hand, D6 was distributed mainly in the context of the stroma of the villi, according to its function of cytoskeleton-dependent decoy scavenger receptor for chemokines (Fig 4).

### 3.5 Placenta inflammation in GDM is independent to BMI

**Fig 5:**
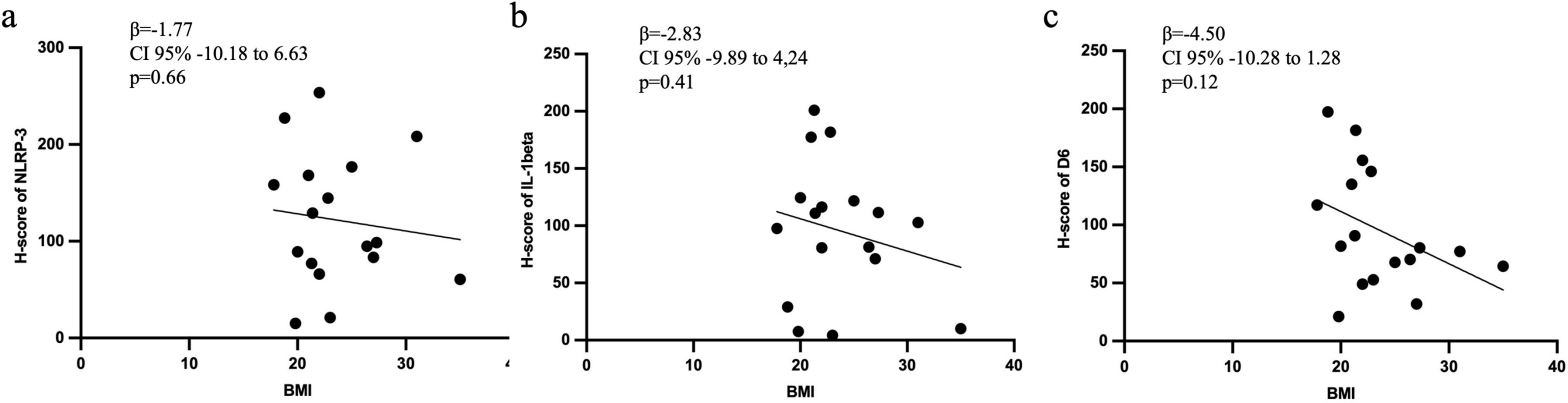
a-c: Scatter plot representing the correlation between placental NLRP-3 (a), IL-1β (b) and D6 (c) expression with maternal BMI. NLRP-3: NLR family pyrin domain containing 3; IL-1β: interleukin 1 beta; CRD6: chemokine scavenger decoy receptor D6; BMI: body mass index.

Linear univariate analysis of the study population for placental assays (n = 17) did not show a significant association between placenta expression of NLRP-3, IL-1β or D6 and maternal BMI (β = −1.77, CI 95% −10.18 to 6.63, p = 0.66; β = −2.83, CI 95% −9.89 to 4,24, p = 0.41;β = −4.50, CI 95% −10.28 to 1.28, p = 0.12, respectively; Fig 5 a and c).

## 4. Discussion

Gestational diabetes mellitus (GDM) represents a significant global health concern due to its growing prevalence and association with adverse maternal-fetal outcomes. In particular, GDM has been associated to a higher risk of preeclampsia, preterm birth and stillbirth [21–23]. Inflammation and immunomodulation at the maternal-fetal interface is essential to support implantation, placental development and function during pregnancy [24,25], thus, any significant inflammatory dysregulation or immune imbalance in that critical site might contribute to the occurrence of obstetric complications [12,26].

In this study, we observed, for the first time, that the placental expression of the inflammasome NLRP-3 is increased in women with GDM at term of pregnancy, compared to normal pregnant women, independently to women’s BMI. Indeed, although the BMI was significantly higher among GDM women compared to normal pregnant women, we could not find a correlation between NLRP-3 placental expression and BMI. We did not find, as expected, higher levels of placental IL-1β but only a trend of increase, probably due to the small sample of patients analyzed. However, higher placental expression of NLRP-3 is consistent with previous observation of increased levels of the toll-like receptor 4 (TLR-4) in GDM placentas [27], a main receptor involved in initiating the assembling of the placental inflammasome NLRP-3. We can speculate that in a condition of insulin-resistance (IR), several extracellular and intracellular stimuli, i.e., free fatty acids (FFA), ionic flux, extracellular ATP (eATP) and ROS may be increased and activate NLRP3 inflammasome and the release of IL-1β [28]. Intriguingly, IL-1β is also the principal inflammatory cytokine implicated in the establishment of IR [28]. In particular, IL-1β has been shown to inhibit insulin signaling in trophoblasts and adipocytes, through the phosphorylation of IRS-1 (insulin receptor substrate 1), implicated in the intracellular cascade of the insulin receptor, and decreasing GLUT-4 (type 4 glucose transporter isoform) expression and glucose uptake [29,30]. Consistently, obese mice knock-out for NLRP-3 show recovering in insulin sensitivity [31], supporting the hypothesis that tissue inflammation and IR are linked in vicious circle of reciprocal amplification.

On the other hand, in this study, placental expression of D6 receptor, which has a pivotal function in maintaining low grade tissue inflammation at maternal-fetal interface, was not found significantly increased in GMD placentas compared to controls. We only observed higher serum levels of the D6-specific cytokines CCL-2 and CCL-4 in GDM women during the third trimester of pregnancy.

While increased serum levels of D6-specific cytokines were detected in GDM women, only a not significant trend of increase in placental D6 expression was found in GDM women compared to controls. This lack of significant difference might be related to the complex intracellular cycling of the receptor in the trophoblast cells. Indeed, once bound to CC chemokines, D6 decoy receptor internalizes and targets the ligands for degradation [32]. While in presence of acute massive inflammation higher levels of D6 receptor would be expected, in a condition of mild chronic inflammation, like in GDM, it is reasonable to detect a less marked increase of placental D6 receptor expression compared to controls.

Serum levels of IFN-γ, a Th-1 pro-inflammatory cytokine, were also increased in GDM women compared to women with normal glucose metabolism, supporting the evidence that GDM women display a mild systemic inflammatory state. Consistently, increased IFN-γ levels have been previously linked to adverse pregnancy outcomes such as GDM, preeclampsia and premature rupture of membranes [33–35].

Indeed, obesity is characterized by chronic low-grade systemic inflammation, mediated primarily by pro-inflammatory markers (e.g., TNF-α) involved in the pathogenesis of insulin resistance and metabolic disorders [36–37].

The main limitations of this study are: a) the small size of the population included; b) the potential confounding effect of higher BMI of the GDM cohort compared to controls on the systemic and placental inflammation. To evaluate the impact of BMI on inflammation, a linear correlation between BMI and serum and placental inflammation-related proteins was performed. Interestingly, only serum CCL-4 levels, but not the other serum or placental cytokines investigated, were associated to BMI, downsizing this potential confounding factor.

In conclusion, not only hyperglycemia, but also systemic and placental inflammation represent a key feature of GDM and a potential pathogenic player in the establishment of related maternal-fetal complications. To our knowledge this is the first study investigating the placental expression of NLRP-3 and D6 in GDM.

Further studies are needed to investigate the role of placental inflammation in the physiopathology of GDM-associated adverse obstetric outcomes and to identify whether new therapeutic strategies able to milden placental and systemic inflammation might improve pregnancy outcome in pregnancies complicated by GDM.

## Supporting information

S1 File Clinical characteristics and obstetric outcomes of women recruited in this study for placental assays. (DOCX)

S1 Data Minimal data set. (XLSX)
