# Supplementary material for: Placental inflammation is increased in gestational diabetes mellitus: The role of inflammasome NLRP-3 and chemokine scavenger decoy receptor D6
Source: PLoS One. 2025 Jun 17;20(6):e0326087. doi: 10.1371/journal.pone.0326087 (PMC12173348; doi:10.1371/journal.pone.0326087)
Supplement: S1 File — (DOCX) [file pone.0326087.s001.docx]

**S1**: Clinical characteristics and obstetric outcomes of women recruited in this study for placental assays.

|  | **GDM (n=10)** | **CTR (n=7)** | **p** |
| --- | --- | --- | --- |
| Age (years) | 36.9 ± 3.7 | 33.3 ± 3.9 | 0.07 |
| BMI (Kg/m2) | 25.6 ± 4.8 | 20.8 ± 1.7 | **<0.05** |
| Race |  |  |  |
| *White* | 7 (70%) | 7 (100%) | - |
| *Hispanic* | 0 (0%) | 0 (0%) | - |
| *Black* | 0 (0%) | 0 (0%) | - |
| *South-East Asian* | 3 (30%) | 0 (0%) | - |
| Nulliparous | 10 (100%) | 6 (85.7%) | - |
| Multiparous | 0 (0%) | 1 (14.3%) | - |
| Previous fetal losses | 2 (20%) | 0 (0%) | - |
| Insulin | 4 (40%) | 0 (0%) | - |
| Diet | 5 (50%) | 0 (0%) | - |
| Metformin | 1 (10%) | 0 (0%) | - |
| GA delivery (weeks) | 38.6 ± 0.5 | 39.1 ± 0.13 | **<0.05** |
| Neonatal Weight (g) | 3440 ± 285.2 | 3670 ± 485.1 | 0.24 |
| Percentile (°) | 73.5 ± 19.9 | 74.6 ± 30.2 | 0.93 |

*Data are expressed as mean ± SD or percentage, according to variables. % refers to the whole cohort of women. BMI: body mass index; GA: gestational age.*
